# Feeding Practice during Diarrheal Episode among Children Aged between 6 to 23 Months in Mirab Abaya District, Gamo Gofa Zone, Southern Ethiopia

**DOI:** 10.1155/2018/2374895

**Published:** 2018-12-02

**Authors:** Teshale Fikadu, Shimels Girma

**Affiliations:** ^1^Department of Public Health, Arba Minch University, Ethiopia; ^2^Department of Psychiatry, Institute of Health, Jimma University, Ethiopia

## Abstract

**Background:**

Diarrheal disease is one of the main causes of childhood malnutrition. In developing countries 30% of pediatric beds are occupied with children having diarrheal disease. Fluid replacement, continued feeding, and increasing appropriate fluid at home during the diarrhea episodes are the cornerstone of treatment package. The purpose of this study was to assess feeding practice during diarrheal episodes among children aged 6 to 23 months in Mirab Abaya district, Gamo Gofa Zone, South Ethiopia.

**Methods:**

Community-based cross-sectional study design was conducted from February to March 2016 among children aged 6 to 23 months. A multistage sampling technique was used to select the study participants. A total of 661 participants were included in our study. Data were entered into Epi data version 3.1 and exported to SPSS 20.0 statistical software for analysis. Bivariate and multivariable analysis were done to assess factors associated with feeding practices during a diarrheal episode. Odds ratio with 95% CI was used to identify a statistically significant association between independent variables and feeding practice during diarrheal episode.

**Result:**

The proportion of proper feeding practice during diarrheal episode was 467 (70.7%). Boy children were about 1.6 times [AOR; 1.62 (95%CI=1.04, 2.50)] more likely to receive increased food and fluid than girl children. Mothers who have one under-five child were 2 times [AOR 2.11 (95% CI =1. 38, 3.23)] more likely to have proper feeding practice during diarrheal episode as compared to those have two and more under-five children. The likelihood of increasing food and fluid during diarrheal episodes was 2 times [AOR 2.46 (95% CI=1. 55, 3.88)] higher among children from maternal age of 30-39 years than those from 20-29 years. Mothers who get information about feeding practices during diarrheal episodes were 2 times [AOR 2.19 (95% CI=1. 43, 3.36)] more likely to increase food and fluid to their child compared to their counterparts.

**Conclusion:**

In this study educational status, number of antenatal care visits, sex, number of under-5 children, maternal age, and mothers information about feeding practice were independently associated with feeding practices during a diarrheal episode. Therefore, intensive intervention programme should focus on these determinants to reduce child mortality and morbidity and realize sustainable development goals.

## 1. Background

Globally, diarrhea is the second leading cause of (19%) under-five deaths, nearly one in five child deaths, about 1.5 million each year, 22.5% of hospitalization, and up to 20% of all outpatient visits in children [1]. Diarrhea is one of the leading causes of childhood morbidity and mortality in developing countries; about 5 million deaths occur each year, 80% of which occur in children less than 2 years [1, 2]. Prevalence and death due to diarrhea were high in low and middle income countries because of low coverage in curative interventions against diarrhea and lack of safe drinking water, sanitation, and hygiene [3, 4]. Ethiopia is one of the five countries with the greatest diarrheal disease burden globally, and 28 % of all under-five hospitalizations in Ethiopia are caused by diarrheal disease [5, 6].

To decrease mortality and morbidity due to diarrheal diseases WHO and UNICEF had laid out a seven-point action plan for comprehensive diarrhea control by the year 2009. Among these points, fluid replacement, continued feeding, and increase appropriate fluids in the home during the diarrhea episode constitute the cornerstone for treatment package [4]. Major portion of death due to diarrheal disease can be prevented by fluid replacement during diarrheal episodes, but only a small proportion of children with life-threatening episodes of diarrhea receive the treatment [7–13]. Fluid replacement with frequent and small feeding including breast feeding during diarrhea episodes has a powerful effect on controlling complication and recovery from diarrhea. Studies showed that 25%, 11%, and 31% of mothers stop breast feeding, decrease fluid administration, and do not give anything during diarrheal episode, respectively [14–16]

In developing countries only 39% and in Africa 35% of children under five with diarrhea receive fluid replacement during diarrheal episodes [4]. Similarly in Ethiopia only 10 % of children with diarrhea were given increased food and fluid replacement [17]. Knowledge about factors associated with feeding practice during diarrheal episode is an important precondition for development of diarrheal disease intervention strategies. Although some studies were conducted in the area and different parts of the country, no previous study was conducted to address feeding practice during diarrheal episode among children of age 6 to 23 months. The aim of this study was to identify feeding practice during diarrheal episode among children of age 6 to 23 months in Mirab Abaya district for the formulation of relevant policy and improvement of implementation and intervention strategy to reduce child mortality and morbidity.

## 2. Methods

### 2.1. Study Design and Setting

A community-based cross-sectional study was conducted in Mirab Abaya district, Gamo Gofa Zone, Southern Ethiopia. The district is located at 400 kilometers south of Addis Ababa (the capital city of Ethiopia) and had a total of 14,784 under-5-year-old children, out of which 2,667 were 6 to 23 months in the year 2016/17, which is projected from 2007 Ethiopia Central-Statistical Agency. In the district there are 4 health centers and 26 health posts providing maternal and child healthcare services.

### 2.2. Source and Study Population

#### 2.2.1. Source Population

All children of age 6 to 23 months living in Mirab Abaya district.

#### 2.2.2. Study Population

All selected children of age 6 to 23 months in Mirab Abaya district found during the study period.

### 2.3. Sample Size Determination and Sampling Procedure

Sample size was calculated using Epi info version 7 statistical software by assuming the expected proportion of mothers who increase food and fluid to their children during diarrheal episode, which is 39.4% [2], with confidence level of 95%, 5% degree of precision, 5% for nonresponse rate, and design effect of 2, and the final sample size was 678. A multistage sampling technique was used. Seventeen kebeles (the smallest administrative unit in Ethiopia) were selected out of 24 kebeles by simple random (lottery method). Then the total sample size was allocated proportionally to the selected kebeles based on the number of children. A list of households with children aged between 6 to 23 months who had diarrheal episode two weeks prior to data collection was used as sampling frame. Then simple random sampling method (generated by computer) was used to select the study participants from each selected kebele. If two or more eligible children were found in the same household, then one of them was selected randomly [18].

### 2.4. Data Collection and Analysis

Pretested structured questionnaire developed by reading different literature was used to collect data. The questionnaire was initially prepared in English, translated into local language, and then retranslated by another person to check consistency. The instrument was pretested in 5% of sample size outside the study kebeles.

Data were collected using structured questionnaire via face to face interviews with mothers/care givers. Twelve diploma nurse data collectors participated during data collection after two days of training. Filled questionnaires were checked daily for their completeness by three supervisors; then they were edited, coded, entered by Epi data version 3.1, and exported to SPSS 20.0 statistical software for analysis. After cleaning data for inconsistencies and missing values, descriptive statistics were done. Then bivariate analysis was done for all explanatory variables to identify association. Variables with p-value less than 0.25 in the bivariate analysis were included in a stepwise backward multivariable logistic regression procedure. Odds ratios (95% confidence intervals) were calculated to determine the association between increased food and fluid during diarrheal episode and independent variables.

In this study diarrhea was measured as passing of three or more loose stools over a period of time that the mother/care giver considered as diarrhea, and proper feeding practice during diarrheal episode was increasing food and fluid, which is measured by four comparative questions on feeding practice during diarrheal episode (frequency of breast feeding before and during the illness, frequency of food intake before and during the illness, amount of food intake before and during the illness, and frequency of fluid intake before and during the illness). If a mother's responses include increase during the illness in ≥ 75% of the total questions, the child is considered as having proper feeding during diarrheal episode (i.e., increased food and fluid).

## 3. Result

A total of 661 participants were included in the study with response rate of 97.5 %. Majority of mothers were Protestant in religion 529 (80%) and house wives in occupation 529 (79.9%). Almost all the respondent mothers were married 650 (98.3%) and about one-third of them had two under-five children in the household 230 (34.8) (Table 1).

All mothers of the study participants were visiting health facility for antenatal care during pregnancy of the index child and all study participants were started vaccination. Mothers of most study participants delivered their children at health facilities 524 (79.3%), got information about feeding practice during diarrheal episode 432 (65.4%), sought medical care during the illness 567 (85.8%), and increased food and fluid during diarrheal episode 467 (70.7%) (Table 2).

### 3.1. Factors Associated with Feeding Practice during Diarrheal Episode

The proportion of those who increased food and fluid during diarrheal episode as recommended were 467 (70.7%). Boy children were about 1.6 times [AOR; 1.62 (95%CI=1.04, 2.50)] more likely to get increased food and fluid than girl children. Mothers who have one under-five child were 2 times [AOR 2.11 (95% CI =1. 38, 3.23)] more likely to have proper feeding practice during diarrheal episode as compared to those who have two and more under-five children. The likelihood of increasing food and fluid during diarrheal episodes was 2 times [AOR 2.46 (95% CI=1. 55, 3.88)] higher among children from maternal age of 30-39 years than those from 20-29 years. Mothers who got information about feeding practices during diarrheal episodes were 2 times [AOR 2.19 (95% CI=1. 43, 3.36)] more likely to increase food and fluid to their children compared to their counterparts (Table 3).

## 4. Discussion

This study intended to identify feeding practice during diarrheal episode among children aged between 6 and 23 months using cross-sectional study design.

The fact of boy preference in developing country has high effect on child care practice; our study supports this idea as mothers of study participants who have boys were 1.6 times more likely to increase food and fluid during diarrheal episode compared to mothers of study participants who have girls. This is consistent with EDHS 2011 report that high proportion of boys receive increased food and fluid during diarrheal episode [17].

Mothers who have one under-five child were 2 times [AOR 2.11 (95% CI =1. 38, 3.23)] more likely to have proper feeding practice during diarrheal episode as compared to those who have two and more under-five children. Our study is comparable with studies conducted in Eastern Ethiopia [2] and Iran [19]. This could be due to the fact that as the number of under-five children increases in the household, the care given to the children decreases, causing strain on family resources, which leads to delay in treatment.

As maternal age increases, exposure to health information and child care practice become better through experience. This study showed that children from mothers of age 30–39 years were 2.46 times more likely to have increased food and fluid compared to children from mothers of age 20–29 years. This is inconsistent with a study in Iran [19]. This may be due to cultural difference. Mothers who get information were 2.2 times more likely to increase food and fluid during diarrheal episode than their counterparts. This is due to the fact that information was an entry point to the development of knowledge.

Increased exposure to health facility increases information related to health promotion and prevention. Those mothers who have four and above ANC follow-ups were 4.21 times more likely to increase food and fluid compared to those who follow ANC less than four times. Also maternal education level affects feeding practice during diarrheal episode. The odds of increasing food and fluid were 2 times and 5 times higher among mothers attending grade 5 to 8 and above 9, respectively. This is consistent with a study conducted in Burkina Faso [20] but it is inconsistent with a study conducted in Ethiopia [2].

## 5. Conclusion

In this study educational status, number of antenatal care visits, sex of child, number of under-5 children, maternal age, and information about feeding practice were independently associated with proper feeding practice during diarrheal episode. Thus, intensifying intervention programmers and stakeholders working on child health should focus on these determinants to reduce child mortality and morbidity and realize sustainable development goals.

## Figures and Tables

**Table 1:** Sociodemographic characteristics for the parent of study participant in Mirab Abaya district, Gamo Gofa Zone, SNNPR, March 2016.

**Variable **	**Number**	**Percent**
Sex		
Male	279	42.2
Female	382	57.8
Age in month		
6 - 12	272	41.1
12 - 18	299	45.2
18 - 24	90	13.6
Number of under 5 year children		
One	431	65.2
Two	230	34.8
Religion		
Protestant	529	80.0
Orthodox	132	20.0
Maternal age in year		
20 - 29	409	61.9
30 – 39	252	38.1
Ethnicity		
Gamo	363	54.9
Wolayta	298	45.1
Marital status		
Married	650	98.3
Single	6	0.9
Divorced	3	0.5
Widowed	2	0.3
Maternal occupation		
Government worker	78	11.8
Student	55	8.3
House wife	528	79.9
Maternal educational status		
No formal education	163	24.7
Grade 1 - 4	136	20.6
Grade 5 - 8	159	24.1
Grade 9 an above	203	30.7

**Table 2:** Health related condition of study population in Mirab Abaya district, Gamo Gofa Zone, SNNPR, March 2016.

Variable	Number	Percent
Place of delivery		
Health institution	524	79.3
Home	137	20.7
Have Information about feeding practice during diarrheal episode		
Yes	432	65.4
No	229	34.6
Frequency of diarrhea in last one month		
1 time	379	57.3
2 time	194	29.3
3 time	88	13.3
Seek medical care during the illness		
Yes	567	85.8
No	94	14.2
Number of ANC follow-ups		
< 4 times	259	39.2
≥ 4 times	402	60.8
Immunization status		
Completed	507	76.7
Up-to-date	154	23.3
Feeding practice during diarrheal episode		
Increase food & fluid	467	70.7
Not increase food & fluid	194	29.3

**Table 3:** Predictors of feeding practice during diarrheal episode in Mirab Abaya district, Gamo Gofa Zone, SNNPR, March 2016.

Variable	Increase food and fluid		COR (95% CI)	AOR (95% CI)
	Yes	No		
	No (%)	No (%)		
Sex				
Male	220(47.1)	59(30.4)	2.04(1.43,2.91)	1.62(1.04,2.50)
Female	247(52.9)	135(69.6)	1	1
Number of under 5 children				
1	327(70.0)	104(53.6)	2.02(1.43,2.85)	2.11(1.38,3.23)
2	140(30.0)	90(46.4)	1	1
Maternal age				
20 - 29	265(56.7)	144(74.2)	1	1
30 – 39	202(43.3)	50(25.8)	2.19(1.52, 3.18)	2.46(1.55, 3.88)
Get information about feeding practice				
Yes	327(70.0)	105(54.1)	1.98(1.40, 2.79)	2.19(1.43, 3.36)
No	140(30.0)	89(45.9)	1	1
Number of ANC follow-ups				
Less than four times	142(30.4)	117(60.3)	1	1
Four and more times	325(69.6)	77(39.7)	3.48(2.45, 4.93)	4.21(2.73, 6.49)
Maternal education				
No formal education	92(19.7)	71(36.6)	1	1
Grade 1 - 4	96(20.6)	40(20.6)	1.85(1.14,2.99)	1.56(0.86, 2.79)
Grade 5 – 8	115(24.6)	44(22.7)	2.02(1.27, 3.21)	1.79(1.04, 3.11)
Grade 9 an above	164(35.1)	39(20.1	3.25(2.04, 5.18)	5.08(2.89, 8.94)

## Data Availability

All relevant data are within the paper.

## Abbreviations

AOR: Adjusted odds ratio
CI: Confidence interval
COR: Crude odds ratio
SNNPR: Southern Nations, Nationalities, and Peoples' Region.

## References

[1] Belachew T., Jira C., Faris K., Mekete G., Asres T., Aragaw H.

[2] Mengistie B., Berhane Y., Worku A. (2013). Prevalence of diarrhea and associated risk factors among children under-five years of age in Eastern Ethiopia. *Open Journal of Preventive Medicine*.

[3] Wardlaw T., Salama P., Brocklehurst C., Chopra M., Mason E. (2010). Diarrhoea: why children are still dying and what can be done. *The Lancet*.

[4] UNICEF (2012). *Pneumonia and Diarrhoea Tackling the Deadliest Diseases for the World’S Poorest Children*.

[5] Mohammed S., Tamiru D. (2013). Morbidity and associated factors of diarrheal diseases among under five children in Arba-Minch district, Southern Ethiopia. *Science Journal of Public Health*.

[6] WHO (2013). *Rotavirus Disease and Vaccines in Ethiopia*.

[7] Mediratta R. P., Feleke A., Moulton L. H., Yifru S., Bradley Sack R. (2010). Risk factors and case management of acute diarrhoea in North Gondar Zone, Ethiopia. *Journal of Health, Population and Nutrition*.

[8] Bhutta Z. A., Das J. K., Walker N. (2013). Interventions to address deaths from childhood pneumonia and diarrhoea equitably: what works and at what cost?. *The Lancet*.

[9] Chopra M., Mason E., Borrazzo J. (2013). Ending of preventable deaths from pneumonia and diarrhoea: An achievable goal. *The Lancet*.

[10] Wilson S. E., Morris S. S., Gilbert S. S. (2013). Scaling up access to oral rehydration solution for diarrhea: Learning from historical experience in low and high performing countries. *Journal of Global Health*.

[11] Gill C. J., Young M., Schroder K. (2013). Bottlenecks, barriers, and solutions: results from multicountry consultations focused on reduction of childhood pneumonia and diarrhoea deaths. *The Lancet*.

[12] Bhutta Z. A., Zipursky A., Wazny K. (2013). Setting priorities for development of emerging interventions against childhood diarrhoea. *Journal of Global Health*.

[13] Mohammed S., Tamiru D. (2014). *The Burden of Diarrheal Diseases among Children under Five Years of Age in Arba Minch District, Southern Ethiopia, and Associated Risk Factors*.

[14] Bani I. A., Saeed A. A. W., Mohammed A. A., Othman A. (2002). Diarrhoea and child feeding practices in Saudi Arabia. *Public Health Nutrition*.

[15] Mohammed S., Tamiru D. (2013). The occurrence of childhood diarhea and its home management among mothers of under-five years children in Arba Minch Zuria, Southern Ethiopia. *Science Journal of Public Health*.

[16] Berhe F., Berhane Y. (2014). Under five diarrhea among model household and non model households in Hawassa, South Ethiopia: A comparative cross-sectional community based survey. *BMC Public Health*.

[17] Central Statistical Agency (CSA) and ICF International (2012). *Ethiopia Demographic and Health Survey 2011*.

[18] Dewana Z., Fikadu T., Facha W., Mekonnen N. (2017). Prevalence and Predictors of Stunting among Children of Age between 24 to 59 Months in Butajira Town and Surrounding District, Gurage Zone, Southern Ethiopia. *Health Science Journal*.

[19] Amini-Ranjbar S., Bavafa B. (2007). Iranian mother's child feeding practices during diarrhea: A study in Kerman. *Pakistan Journal of Nutrition*.

[20] Wilson S. E., Ouédraogo C. T., Prince L. (2012). Caregiver Recognition of Childhood Diarrhea, Care Seeking Behaviors and Home Treatment Practices in Rural Burkina Faso: A Cross-Sectional Survey. *PLoS ONE*.

